# Diverse Components of Resistance to *Fusarium verticillioides* Infection and Fumonisin Contamination in Four Maize Recombinant Inbred Families

**DOI:** 10.3390/toxins11020086

**Published:** 2019-02-01

**Authors:** Laura Morales, Charles T. Zila, Danilo E. Moreta Mejía, Melissa Montoya Arbelaez, Peter J. Balint-Kurti, James B. Holland, Rebecca J. Nelson

**Affiliations:** 1 School of Integrative Plant Science, Cornell University, Ithaca, NY 14853, USA; dem324@cornell.edu (D.E.M.M.); mmontoyaa@unal.edu.co (M.M.A.); 2 Department of Crop and Soil Sciences, North Carolina State University, Raleigh, NC 27695 USA; ctzila@ncsu.edu (C.T.Z.); james_holland@ncsu.edu (J.B.H.); 3 Department of Entomology and Plant Pathology, North Carolina State University, Raleigh, NC 27695, USA; pjbalint@ncsu.edu; 4 Plant Science Research Unit, United States Department of Agriculture–Agricultural Research Service, Raleigh, NC 27695, USA

**Keywords:** maize, mycotoxins, fumonisin, disease resistance, morphology

## Abstract

The fungus *Fusarium verticillioides* can infect maize ears, causing Fusarium ear rot (FER) and contaminating the grain with fumonisins (FUM), which are harmful to humans and animals. Breeding for resistance to FER and FUM and post-harvest sorting of grain are two strategies for reducing FUM in the food system. Kernel and cob tissues have been previously associated with differential FER and FUM. Four recombinant inbred line families from the maize nested associated mapping population were grown and inoculated with *F. verticillioides* across four environments, and we evaluated the kernels for external and internal infection severity as well as FUM contamination. We also employed publicly available phenotypes on innate ear morphology to explore genetic relationships between ear architecture and resistance to FER and FUM. The four families revealed wide variation in external symptomatology at the phenotypic level. Kernel bulk density under inoculation was an accurate indicator of FUM levels. Genotypes with lower kernel density—under both inoculated and uninoculated conditions—and larger cobs were more susceptible to infection and FUM contamination. Quantitative trait locus (QTL) intervals could be classified as putatively resistance-specific and putatively shared for ear and resistance traits. Both types of QTL mapped in this study had substantial overlap with previously reported loci for resistance to FER and FUM. Ear morphology may be a component of resistance to *F. verticillioides* infection and FUM accumulation.

## 1. Introduction

In maize (*Zea mays* L), the fungus *Fusarium verticillioides* causes Fusarium ear rot (FER) and contaminates grain with fumonisins (FUM), a family of mycotoxins produced by *Fusarium* spp. [1]. FER and FUM contamination can reduce yields and grain marketability [2]. FUM exposure has also been linked to adverse health outcomes in humans and domesticated animals [3,4,5,6,7,8]. The combination of the role of maize as a staple crop, conducive environmental conditions for *F. verticillioides* infection (FVI), and limited regulation of mycotoxins in food systems has led to high FUM exposure in the developing world [4,5,6,9,10,11]. 

Pre- and post-harvest management can reduce FUM levels in food systems. Agronomic practices, such as crop residue removal and soil nutrient management, have been associated with reduced risk of FUM contamination in maize [12,13]. Breeding for resistance to FVI and FUM contamination in maize varieties is a more long-term pre-harvest approach [14]. Post-harvest strategies include visual and optical grain sorting [15,16] and thermochemical treatments [17,18].

Genetic resistance to FER and FUM contamination in maize is moderately to highly heritable and quantitatively controlled, making breeding for durable resistance feasible [19,20,21,22]. Both morphological and biochemical characteristics have been shown to contribute genetic resistance. Biochemical signatures associated with differential FVI and FUM levels include lipid profiles [23] and biosynthesis of oxylipins and plant hormones, such as ethylene, abscisic acid, salicylic acid, and jasmonic acid [24,25]. The phenylpropanoid pathway, which is involved in lignin biosynthesis, has also been implicated in resistance to FVI and FUM contamination, although it is unknown whether phenylpropanoids play a passive role in resistance via pericarp hardening or whether they have an active inhibitory effect on *F. verticillioides* [24,26,27].

Cob and kernel morphological traits have been linked to differential FVI severity and FUM contamination. FVI and FUM levels have been negatively associated with kernel traits related to density and hardness [26,28,29,30,31,32,33], and kernel bulk density (also known as test weight) under *F. verticillioides* inoculation has been shown to be genetically correlated with FER and FUM [33,34]. Resistance to FUM contamination could therefore hypothetically be accomplished via indirect selection for increased kernel bulk density [35]. Cob morphology, an important component of yield [36], has also been shown to be genetically correlated with FER and FUM [33]. Variation in external symptomatology of kernels infected with *F. verticillioides* has been associated with differential FUM accumulation [33,37,38,39], suggesting that qualitative measures of kernel symptom severity are important components of resistance.

Understanding the variation in and the role of the maize host genetics in symptomatology of kernels under *F. verticillioides* inoculation could potentially reveal diverse modes of pathogenesis and/or host resistance. The maize nested association mapping (NAM) population, composed of 25 recombinant inbred line (RIL) families derived from 25 diverse inbred lines crossed to one recurrent inbred parent (B73), is a powerful tool for dissecting the genetic architecture of quantitatively inherited traits [40]. Here we employed quantitative, qualitative, external, and internal indicators of FVI severity as well as publicly available data on ear architecture phenotypes [41,42] to dissect the genetic mechanisms underlying FVI-specific and ear-mediated resistance to FVI and FUM accumulation in four NAM RIL families.

## 2. Results

### 2.1. Symptomatology Varies among Families

Four NAM RIL families (B73 × CML333, B73 × CML52, B73 × CML69, and B73 × NC358) were grown in an augmented incomplete block design across four environments. We inoculated the ears with toothpicks coated in *F. verticillioides* spores, a method that has previously been shown to reveal cob and kernel resistance mechanisms [33]. We evaluated the inoculated ears for external and internal indicators of disease severity. External phenotypes included FER (proportion of visibly infected kernels) and a symptom typology to account for qualitative variation in FER. Internal measures were grain FUM concentration, grain toxin load per visibly infected area (FUM:FER), and kernel bulk density (BDEN_inoc_). As demonstrated by ANOVA, all four quantitative FVI indicators (FER, FUM, FUM:FER, BDEN_inoc_) differed significantly among families (Table 1). On average, families that had higher BDEN_inoc_ tended to have lower FER, FUM and FUM:FER (Table 1). The B73 × CML333 family was unique in that it had high FER scores but had the least severe internal disease severity (highest BDEN_inoc_ and low FUM and FUM:FER) (Table 1). 

**Figure 1:**
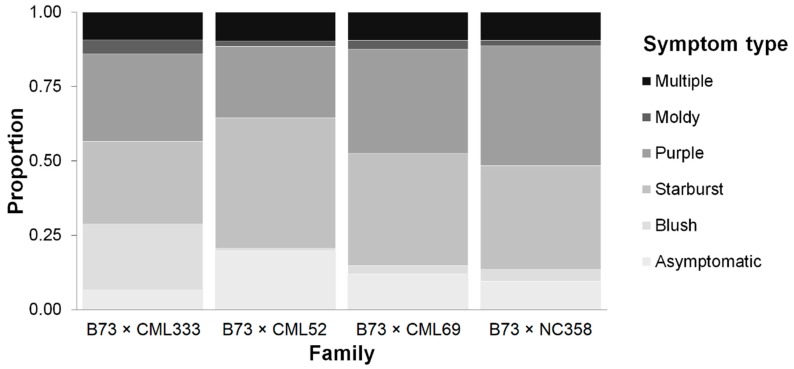
Proportions of plots exhibiting distinct symptom types in four RIL families across three environments.

The composition of symptom types significantly differed among families, as determined by a *χ*^2^ test comparing the proportions of plots exhibiting asymptomatic, blush, starburst, purple, moldy, or multiple symptom types across three environments among the four RIL families (*χ*^2^ = 265, *p* < 0.0001). Across families, the majority of plots (57% to 75%) exhibited the starburst or purple symptom types (Figure 1). The B73 × CML333 family had the greatest proportion of plots exhibiting the blush type (22%) (Figure 1). The B73 × CML52 family had the largest proportion of asymptomatic (20%) and starburst (44%) plots (Figure 1). The proportion of purple plots was greatest in the B73 × CML69 (35%) and B73 × NC358 (40%) families (Figure 1). Approximately 9% of plots exhibited multiple symptoms in all families (Figure 1). 

### 2.2. Relationships among External Symptomatology, Kernel Bulk Density, and Toxin Load Vary among Families

BDEN_inoc_ differed significantly among symptom types in all analyses (ANOVA, *p* < 0.0001) (Table 2). Pairwise *t*-tests demonstrated that greater external symptom severity was associated with more greatly reduced BDEN_inoc_ in all families (Table 2). Specifically, asymptomatic plots had the highest BDEN_inoc_, followed by blush, starburst, purple, and moldy plots in descending order (Table 2).

FER and FUM:FER differed significantly among symptom types in all analyses, but pairwise differences between symptom types varied by family (Table 2). Similarly, symptom types differed significantly with respect to FUM in the combined-family, B73 × CML333, and B73 × CML69 analyses, but symptom severity was not clearly linked to FUM contamination (Table 2). Unexpectedly, asymptomatic plots had the highest or second-highest FUM contamination across all families (Table 2). This phenomenon may be the result of the symptom type scoring method, which was conducted with the kernels still attached to the cob and thus kernels may have had symptoms present below the visible area.

### 2.3. Fusarium Ear Rot Severity, Fumonisin Contamination, and Kernel Bulk Density are Correlated

We assessed correlations among indicators of FVI severity and FUM contamination at the levels of plot phenotype and genotype means (after controlling for genetic and field effects). Across all families, FUM and FER were (1) positively phenotypically and/or genotype-means correlated with each other and (2) negatively phenotypically and genotype-means correlated with BDEN_inoc_ (Table 3). These correlations are consistent with those previously reported [33,35,43,44]. FUM tended to be more strongly correlated with BDEN_inoc_ than with FER across families (Table 3), suggesting that internal infection severity (as reflected by BDEN_inoc_) may be a better indicator of FUM contamination that external symptom severity (FER).

### 2.4. Innate Ear Morphology is a Component of Resistance to FVI and FUM Contamination

Previous studies have reported associations between FVI severity and FUM contamination with characteristics of the cob and kernel tissues, such as cob morphology and kernel density-related traits [28,32,33,34,45,46,47]. Given the symptomatological variation present in the four RIL families, we sought to test the extent to which innate (uninoculated) ear architecture played a role in resistance to FVI using publicly available data on cob density (CobDen), diameter (CobDiam), length (CobLen), mass (CobMass), and volume (CobVol) as well as uninoculated kernel bulk density (BDEN_uninoc_).

Supporting the hypothesis that maize lines with greater innate kernel bulk density (BDEN_uninoc_) in turn have greater bulk density under *F. verticillioides* inoculation (BDEN_inoc_), we found that BDEN_uninoc_ and BDEN_inoc_ were positively correlated in all families (Table S1). BDEN_uninoc_ was negatively correlated with FER in the B73 × CML52 and B73 × CML69 families and positively correlated with FUM:FER in the B73 × CML333 and B73 × CML52 families (Table S1). BDEN_uninoc_ was less strongly correlated with FER and FUM:FER than was BDEN_inoc_ (Table 3 and Table 4). Unlike the correlations found between FUM and BDEN_inoc_, FUM was not significantly (*p* > 0.05) correlated with BDEN_uninoc_ in any family (Table S1). 

Although the statistical significance of correlations between cob size traits and BDEN_inoc_ or BDEN_uninoc_ varied among families, the directionality of these correlations was generally conserved across families (Table S1). Both BDEN_inoc_ and BDEN_uninoc_ tended to be negatively correlated with CobDiam, CobMass, and CobVol (Table S1). Relationships between cob morphology and FER, FUM, and FUM:FER varied from family to family. In general, lines with larger cobs had greater FER severity, as demonstrated by positive correlations between FER and CobDiam or CobVol across families (Table S1). FER was negatively correlated with CobDen in the B73 × CML52 family and positively correlated with CobMass in the B73 × NC358 family (Table S1). FUM:FER correlations with cob size and/or density in the B73 × CML52 and B73 × NC358 families revealed that lines with larger, less dense cobs had reduced FUM:FER (Table S1). We expected to find similar relationships between FUM and cob morphology, but instead found that FUM was only marginally positively correlated with CobMass in the B73 × CML52 family. This may have been due to the low genetic variation for FUM in this study. Similar to previous studies [33,36], cob size traits (CobDiam, CobLen, CobVol) were significantly (*p* < 0.05) or marginally significantly (*p* < 0.1) positively correlated with each other and with CobMass. CobDen was significantly positively correlated with CobMass and significantly or marginally significantly negatively correlated with cob size traits (Table S1). 

### 2.5. Genetic and Environmental Variation on Disease Severity Differ among Families 

**Figure 2:**
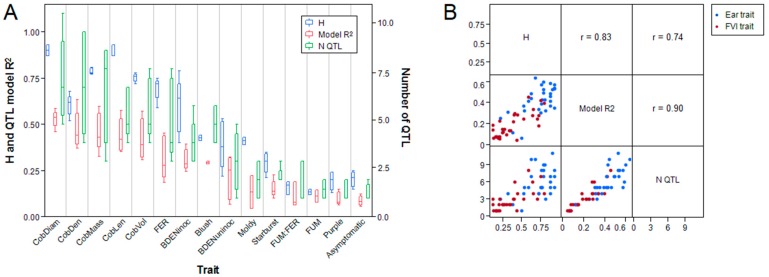
(**A**) Box-plots comparing H, number of associated quantitative trait loci (QTL), and variance explained by QTL models (model *R*^2^) among BDEN_inoc_, FER, FUM, and the ratio of FUM to FER (FUM:FER) under *F. verticillioides* inoculation, and kernel bulk density (BDEN_uninoc_) and cob density, diameter, length, mass, and volume under uninoculated conditions. (**B**) Pearson correlations among H, number of associated QTL, and QTL model *R*^2^ for the previously described *F. verticillioides* infection (FVI) and ear traits (*p* < 0.0001 for all three pairwise correlations).

We assessed genetic and environmental effects on indicators of FVI severity within and among families. Across all families, broad-sense heritability of genotype-means (H) was greater for BDEN_inoc_ (0.4–0.79) and FER (0.59–0.75) than for FUM (0.12–0.17), FUM:FER (0.12–0.21), or symptom type traits (0–0.44) (Table 4, Figure 2). The variance explained by genotype, environment and the interaction between genotype and environment (G*E) varied widely by trait and family (Table 4). Of the nine FVI traits, the moldy symptom type was most affected by G*E (68% to 85% variance in all families) (Table 4). In general, G*E explained less than 20% of the variance in all other traits across families (Table 4). Traits related to fumonisin contamination (FUM and FUM:FER) had lower genetic variance (1% to 3%) and higher variance explained by environment (46% to 73%) than the other traits across all families (Table 4). In contrast, BDEN_inoc_ and FER had the highest genetic variance (BDEN_inoc_ = 23% to 64%; FER = 22% to 42%), and environment explained the least amount of variance in BDEN_inoc_ (0.3% to 8%) and the moldy symptom type (1% to 3%) (Table 4). The H for FUM found in our experiment was much lower than those previously reported (0.43–0.86) [43,44]. Resistance to mycotoxigenic fungi and kernel composition are influenced by environmental conditions and management practices [13,19,36,37,48,49,50,51], which may explain the large environmental and G*E effects here.

### 2.6. Genetic Architecture Differs between Ear Morphological and Resistance Traits

In total, stepwise regression selected 271 quantitative trait loci (QTL) associated with the nine FVI traits and the six ear traits in the single and joint family models from the total set of 7386 markers (Table S2). Sixty-six QTL were selected in the joint family models, and 57, 41, 59, and 48 QTL were mapped in the B73 × CML333, B73 × CML52, B73 × CML69, and B73 × NC358 families, respectively (Table S2). Traits that had greater H also had a greater number of associated QTL and greater variance explained by their respective QTL models (model *R*^2^) (Figure 2). Cob morphological traits tended to have a greater number of associated QTL (28–37 QTL each) and QTL model *R*^2^ (model *R*^2^ = 0.31–0.63) than BDEN_uninoc_ and FVI traits (3–15 QTL, *R*^2^ = 0.06–0.32), with the exception of BDEN_inoc_ (20 QTL, model *R*^2^ = 0.28–0.39), FER (26 QTL, *R*^2^ = 0.18–0.45), and the blush symptom type (10 QTL, model *R*^2^ = 0.29–0.30) (Figure 2).

### 2.7. Most QTL for Resistance to F. verticillioides are Trait-Specific

Within families, the majority of QTL were specific to one trait (161/271 QTL) (Table S3). A total of 110 QTL overlapped with QTL for at least one other trait within families at 44 distinct regions (“colocalized QTL”) (Table S3). Twenty-one colocalized QTL were associated with multiple ear traits but not associated with any FVI trait (Table S3). Ten colocalized QTL were associated with multiple FVI traits but no ear traits (“FVI-specific colocalized QTL”), with three each from the B73 × CML69 and B73 × CML333 families, two from the B73 × NC358 family, and one each from the B73 × CML52 and joint family analyses (Table 5). Thirteen colocalized QTL were associated with both FVI and ear traits (“FVI-ear colocalized QTL”), with five from the joint analysis, three from the B73 × CML52 family, three from the B73 × CML333 family, and one each from the B73 × CML69 and B73 × NC358 families (Table 5). 

### 2.8. Allele Effects at FVI-Specific Loci Reflect Trait Relationships

The directionality of allele effects on quantitative FVI traits (e.g., FER, FUM) that shared QTL with each other and/or with BDEN_uninoc_ generally matched the phenotypic correlation between the corresponding traits. For example, FER was negatively correlated with both BDEN_inoc_ and BDEN_uninoc_ in the B73 × CML52 and B73 × CML69 families (Table 3 and Table 5), and allele effects on FER were opposite to allele effects on BDEN_inoc_ and BDEN_uninoc_ at three colocalized QTL in the B73 × CML52, B73 × CML69, and joint family analyses (Table 5). Similarly, FER was positively correlated with FUM and negatively correlated with FUM:FER across all families (Table 3 and Table 5), and the allele effect on FER was in the same direction as the allele effect on FUM at a colocalized QTL in the B73 × CML333 family and in the opposite direction as the allele effect on FUM:FER at colocalized QTL in the B73 × CML333 and B73 × CML52 families (Table 5). 

QTL colocalized for multiple symptom type traits generally had opposite allele effects on mild (e.g., blush, starburst) vs. severe (e.g., purple, moldy) symptom types (Table 5), indicating that related mechanisms may underlie the manifestation of similar symptomatologies (blush/starburst or purple/moldy). For example, allele effects on starburst were in the same direction as those on blush and opposite to allele effects on moldy and purple at colocalized QTL (Table 5). Similarly, asymptomatic lines by definition have low FER, and allele effects on FER were opposite to asymptomatic allele effects at their two colocalized QTL (Table 5). 

### 2.9. Characteristics of Loci Underlying Specific and Ear-Mediated Resistance to F. verticillioides

We sought to characterize and compare FVI-specific and FVI-ear QTL. First, we tested whether the two classes of resistance loci differed with respect to marker effect size, controlling for trait, family, and QTL length effects. We found that trait and family were significantly *(p <* 0.0001) and marginally significantly *(p <* 0.1) associated with absolute marker effect size, respectively. As the trait scale for FER and CobVol was larger than the other FVI and ear traits, these two traits in turn had larger absolute marker effects. Compared to the other families, the B73 × NC358 family had greater marker effect sizes and the joint family models had smaller marker effect sizes. We then investigated whether FVI-specific and FVI-ear loci tended to be found in pericentromeric regions, which have lower recombination rates than non-centromeric regions of the genome [52]. The FVI-specific (10/62 QTL) and FVI-ear (4/13 QTL) groups did not significantly differ with respect to the proportion of QTL located in pericentromeric bins (*χ*^2^ = 1.5, *p* = 0.2). Several other studies have mapped loci for resistance to *F. verticillioides*, and we identified which of our FVI-specific and FVI-ear QTL colocalized with previously described FVI loci [19,49,53,54,55,56,57,58,59]. A significantly greater proportion of our FVI-specific QTL (46/62 QTL) overlapped published FVI loci than did our FVI-ear QTL (6/13) (*χ*^2^ = 4.0, *p* = 0.04). 

## 3. Discussion

Here we dissect the diversity of genetic resistance to FVI and FUM accumulation in four maize RIL families. We further establish the importance of BDEN_inoc_ as a useful proxy for FUM [33,34], especially given the poor heritability of FUM contamination in maize grain. In addition, we demonstrate that some loci underlying resistance FVI and FUM severity may have trade-offs for agronomically important kernel and cob traits. 

At the phenotypic, genetic, and locus levels, BDEN_inoc_ was negatively associated with external symptom severity and FUM contamination. These results strengthen the arguments that BDEN_inoc_ could be used as a predictor of FUM contamination [28,32,33,34] and that selection for increased BDEN_inoc_ could indirectly increase resistance to FUM [35], even under conditions resulting in low heritability for FUM. The relatively low heritability for FUM in our experiment may have been due to the inoculation method (insertion of spore-coated toothpick into the developing ear), which has been shown to be less effective for screening genetic sources of resistance to FUM contamination [33,60]. However, toothpick inoculation, which has been shown to be effective at screening ear-mediated resistance mechanisms [33], allowed us to study the role of the cob in resistance to FVI and FUM. We identified 23 novel QTL associated with resistance to FVI, seven of which may be mediated by kernel and cob architecture.

Although no FUM or FUM:FER QTL colocalized with ear QTL in this study, we did find that lines with larger, less dense cobs had greater FER and lower BDEN_inoc_ at the QTL and trait correlation levels. In addition, both FER and BDEN_inoc_ were phenotypically and genetically correlated with FUM here and in previous studies [33,34] and we found substantial overlap between our ear QTL and previously described loci for resistance to FUM [19,49,53,54,55,56,57,58,59], suggesting that there may indeed be a genetic link between cob morphology and FUM but that the low heritability for FUM in our experiment may have limited our ability to detect this inferred relationship. 

As cob morphology and kernel density are important components of grain yield [36] and quality [61], respectively, increased susceptibility to FVI and FUM contamination may be a by-product of the breeding process. We are currently conducting follow-up experiments with near isogenic lines (NILs) to dissect possible pleiotropy between ear architecture and resistance to FVI and FUM. We also recommend that kernel bulk density under *F. verticillioides* infection be more thoroughly evaluated as a proxy for FUM contamination in maize grain. The wide variation in internal vs. external symptomatology, inter-trait correlations, and QTL colocalizations in this study implies that distinct modes of resistance exist within tropical germplasm, which can be leveraged for resistance breeding.

## 4. Materials and Methods

### 4.1. Field Design and Inoculation

Four RIL families (B73 × CML333, B73 × CML52, B73 × CML69, B73 × NC358) from the maize NAM population [40] and the five inbred parents (B73, CML333, CML52, CML69, NC358) were grown at the Central Crops Research Station in Clayton, NC in four year-environments from 2012 to 2015. Each NAM family is composed of 200 RILs. The four non-B73 parents originate from breeding programs in Mexico and North Carolina—regions that are prone to mycotoxin contamination [5,62]—and have previously demonstrated greater resistance to FER than B73 [20,21]. We used an augmented incomplete block design, in which each family was grown separately with 20-plot blocks. RIL plots were replicated once per year-environment and randomized within family, and the two parental lines of each family were randomized in each 20-plot block. All RILs from the B73 × CML52 and B73 × NC358 families were grown in four year-environments from 2012 to 2015. For the B73 × CML333 and B73 × CML69 families, all RILs from these two families were grown in three year-environments from 2013 to 2015 and 40 randomly selected RILs from each of these two families were grown in 2012. We measured days to silking (DTS) in each plot as described by Buckler et al. [63]. One toothpick coated with spores from local toxigenic *F. verticillioides* isolates was inserted into the middle of each developing primary ear approximately 10 days after silking, and the toothpick remained in the ear until harvest [21].

### 4.2. Disease Phenotyping

Primary ears were harvested from each plot at maturity, dried, and then visually evaluated for FER. FER was scored based on the percentage of the kernels presenting symptoms on a 1% to 100% scale with 5% increments [19]. The average FER score of all the ears in each plot was then calculated. 

We used a symptom typology to account for the qualitative variation in external FER symptoms, in which each ear was assigned a symptom type: “asymptomatic,” “blush,” “starburst,” “purple,” or “moldy” (photographic examples in Morales et al. [33]). Asymptomatic ears had no or very limited visible external symptoms [33,38,39]. The blush type was characterized by pink discoloration localized on the kernel crowns [33]. Starburst ears had whitish streaks radiating from the kernel silk scar and/or pedicel [33,38,64,65]. The kernels of purple type ears were visibly degraded and had severe purplish discoloration [33]. Moldy ears had severely degraded kernels with matted fungal growth [33,37,38]. Each plot was assigned a main symptom type based on the most frequent symptom type of the ears in the plot. Plots with less than 10% average FER were considered asymptomatic. Plots with equal representation of more than one symptom type were categorized as “multiple.”

After scoring FER and symptom typology, ears were then shelled and bulked per plot. From each plot, a random 250-mL volume of kernels was weighed, and kernel bulk density (BDEN_inoc_) was calculated as the weight of the kernels divided by 250 mL. After weighing, the kernels were returned to the bulked plot.

All of the kernels in each bulked plot were ground into a fine powder with a Waring 7010 two-speed laboratory blender (Waring Commercial, Inc., Torrington, CT, USA). A 10-g subsample from each ground bulk was put in a 25 mL centrifuge tube. To extract fumonisins, 20 mL of 90% methanol was added to each 25 mL tube, resulting in a two-fold dilution factor at this step. The tubes were then shaken with a Lab-Line Environ Orbitol Shaker (Lab-Line Instruments, Inc., Melrose Park, IL, USA) at 150 rpm for approximately five minutes. The samples settled for 15 min, after which 0.5 mL of supernatant from each sample was transferred to a 15 mL centrifuge tube. To dilute the supernatant to a final 40-fold dilution, 9.5 mL of distilled water was added to each 15 mL tube. Fumonisin contamination (FUM) was quantified with FUM-specific enzyme-linked immunosorbent assay (ELISA) kits (Helica Biosystems, Inc., Santa Ana, CA, USA). Absorbance at 450 nm of the ELISA plates was read using a BioTek µQuant™ microplate spectrophotometer (BioTek Instruments, Inc., Winooski, VT, USA) paired with Gen5™ software (BioTek Instruments, Inc., Winooski, VT, USA). Samples that had FUM levels predicted to be above the highest standard provided by the ELISA kits, 6 µg g^−1^ (ppm), were serially diluted until their predicted FUM levels were within the standard curve. To approximate samples that had non-detectable FUM levels (<0.1 ppm), uniform random values between 0 and 0.1 ppm were assigned to these samples [66]. The ratio of FUM to FER (FUM:FER) was calculated as FUM/(FER+1).

### 4.3. Mixed Models and Heritability Estimation

For mixed model analysis, we treated each of the five symptom types as absence/presence traits. Each plot was assigned as having the absence (0) or presence (1) of each symptom type. For example, if a plot exhibited the blush symptom type, it was assigned 1 for blush and 0 for asymptomatic, starburst, purple, and moldy. As the raw BDEN_inoc_, FER, FUM, and FUM:FER data were not normally distributed, they were Box-Cox transformed using JMP^®^software (SAS Institute Inc., Cary, NC, USA, 1989–2007) for further analysis. 

We used JMP^®^software (SAS Institute Inc., Cary, NC, USA, 1989–2007) to fit mixed linear models for the five symptom type absence/presence traits and the four quantitative traits (Box-Cox-transformed BDEN_inoc_, FER, FUM, and FUM:FER). Within and among families, mixed models were fit for each of the nine FVI traits as a separate response variable with environment, block[environment], genotype, and genotype*environment as random effects and DTS as a fixed covariate. Variance components from each model were extracted and used to calculate broad-sense heritability (H) as

$$\frac{\sigma_{G}^{2}}{\sigma_{G}^{2}+\frac{\sigma_{GE}^{2}}{e_{h}}+\frac{\sigma_{\varepsilon }^{2}}{p_{h}}},$$

where *σ^2^_G_*, *σ^2^_GE_*, and *σ^2^_ε_* are the genotype, genotype*environment, and error variances, respectively, and *e_h_* and *p_h_* are defined as

$$e_{h}=\frac{n}{\sum_{i=1}^{n}\frac{1}{e_{i}}},$$

$$p_{h}=\frac{n}{\sum_{i=1}^{n}\frac{1}{p_{i}}},$$

where *n* is the number of genotypes and *e_i_* and *p_i_* are the number of environments and plots for the *i*^th^ genotype, respectively [67]. 

We also sought to assess associations between FVI and kernel bulk density (BDEN_uninoc_) and cob morphology under non-inoculated conditions. Total kernel volume, cob diameter (CobDiam), cob length (CobLen), cob mass (CobMass), and ear mass had been previously measured in the NAM in five location-environments in 2006 (Aurora, NY, USA; Clayton, NC, USA; Homestead, FL, USA; Ponce, Puerto Rico; Urbana, IL, USA) [41,42], and these publicly accessible data for our four NAM families were provided by Panzea [42]. BDEN_uninoc_ was calculated as (ear mass—CobMass)/total kernel volume. Assuming cob shape to be cylindrical, cob volume (CobVol) was calculated as π*CobLen*(CobDiam/2)^2^. Cob density (CobDen) was calculated as CobMass/CobVol. To estimate genotype means for BDEN_uninoc_, CobDen, CobMass, and CobVol, we fit mixed linear models within and among families for these four ear traits with genotype and environment as random effects and DTS as a fixed effect using JMP^®^software (SAS Institute Inc., Cary, NC, USA, 1989–2007). Broad-sense heritability (H) was calculated as

$$H=\frac{\sigma_{G}^{2}}{\sigma_{G}^{2}+\frac{\sigma_{\varepsilon }^{2}}{e}},$$

where *e* is the number of environments and σ^2^_G_ and σ^2^_ε_ are the genotype and error variances, respectively. Genotype means and broad-sense heritability for CobDiam and CobLen were accessed from Brown et al. [41].

### 4.4. Trait Correlation Analyses

Pairwise Pearson correlations among plot phenotypes and among trait best linear unbiased predictors (BLUPs) for quantitatively measured traits (BDEN_inoc_, BDEN_uninoc_, FER, FUM, FUM:FER) were calculated within families using JMP^®^software (SAS Institute Inc., Cary, NC, USA 1989–2007). Both plot-level (phenotypic) and BLUP (genetic) correlations were assessed among the FVI traits. Genetic correlations among BLUPs for BDEN_uninoc_ and FVI traits were also calculated.

### 4.5. Comparison of Disease Severity among Families

We tested differences in FVI severity among families using JMP^®^software (SAS Institute Inc., Cary, NC, USA, 1989–2007). We used analysis of variance (ANOVA) to test the effect of family on BDEN_inoc_, FER, FUM, and FUM:FER, and pairwise two-tailed *t*-tests to asses differences in these four traits between families. A likelihood ratio *χ*^2^ test was used to compare the composition of symptom types among families.

### 4.6. QTL Mapping, Colocalization, and Allele Effects

The NAM population had been analyzed with genotyping-by-sequencing (GBS) technology [68]. A subset of 7386 GBS markers with 0.2 cM resolution had been selected by Olukolu et al. [69]. We extracted the 7386 GBS markers for the B73 × CML333, B73 × CML52, B73 × CML69, and B73 × NC358 NAM families and used them for stepwise regression.

Stepwise regression models were fit in TASSEL version 5.2.37 [70] to identify markers associated with BDEN_inoc_, BDEN_uninoc_, FER, FUM, FUM:FER, and the five symptom types (asymptomatic, blush, starburst, purple, moldy). For the single-family models, a marker significance threshold of 0.001 was used [71]. For the joint-family models, marker effects were nested within family and the significance threshold was set to 0.0001 [71]. QTL were defined by the confidence intervals (CIs) of markers selected by stepwise regression [70]. Within each family, QTL were considered colocalized if their CIs overlapped. 

In addition to identifying colocalized QTL, we also sought to investigate allele effect relationships at specific colocalized QTL. We extracted allele effect estimates from the TASSEL stepwise regression model outputs [70] and then compared allele effects on traits that had colocalized QTL. 

### 4.7. Characterization of Resistance QTL

We classified the QTL mapped in our study as (1) FVI-specific if the trait-specific or colocalized QTL was only associated with FVI trait(s) or (2) FVI-ear colocalized if the QTL was associated with both ear and FVI trait(s). With JMP^®^software, we fit a linear model including absolute marker effect as the response and trait, family, resistance locus type, and QTL confidence interval size as fixed effects. We determined in which genetic bin(s) each resistance QTL was located using bin physical position (AGPv2) information from MaizeGDB [72], and if the QTL overlapped the bin containing the centromere on the respective chromosome, the QTL was classified as pericentromeric. We then compared the proportion of pericentromeric QTL between the two classes of resistance loci. We amassed loci associated with resistance to *F. verticillioides* from nine publications [19,49,53,54,55,56,57,58,59] and determined which of our QTL overlapped with said previously described loci.

## Figures and Tables

**Table 1:** Comparison of kernel bulk density (BDEN_inoc_), Fusarium ear rot (FER), fumonisin concentration (FUM), and the ratio of FUM to FER (FUM:FER) under *Fusarium verticillioides* inoculation among four nested association mapping (NAM) recombinant inbred line (RIL) families and among the five parental inbred lines. Analyses included (1) ANOVA for BDEN_inoc_, FER, FUM, and FUM:FER vs. family or parent, and (2) pairwise *t*-tests comparing BDEN_inoc_, FER, FUM, and FUM:FER between families or parents. FER was evaluated across four environments, FUM and FUM:FER across three environments, and BDEN_inoc_ across two environments.

Family	RILs (N/env)	BDEN_inoc _*** (g mL^−1^)	FER*** (%)	FUM*** (ppm)	FUM:FER *** (ppm %^−1^)
B73 × CML333	186	0.737 ± 0.003 A	43.04 ± 1.27 A	24.24 ± 5.32 B	0.51 ± 0.10 C
B73 × CML52	177	0.720 ± 0.004 B	34.41 ± 1.05 B	11.77 ± 2.39 B	0.60 ± 0.08 B
B73 × CML69	186	0.712 ± 0.003 B	40.04 ± 1.23 A	31.28 ± 5.05 A	1.00 ± 0.13 AB
B73 × NC358	179	0.683 ± 0.004 C	43.27 ± 1.20 A	32.62 ± 4.21 A	1.19 ± 0.23 A
**Parent**	**Plots (N/env)**	BDEN_inoc _*** **(g mL^−1^)**	FER*** **(%)**	FUM*** **(ppm)**	**FUM:FER** **(ppm %^−1^)**
B73	39	0.650 ± 0.005 C	47.83 ± 2.26 A	72.93 ± 14.41 A	2.36 ± 0.64 A
CML333	11	0.770 ± 0.008 A	26.65 ± 3.37 B	7.09 ± 2.82 B	0.47 ± 0.18 B
CML52	11	0.714 ± 0.020 B	13.39 ± 3.17 C	3.18 ± 0.78 B	0.76 ± 0.26 AB
CML69	11	0.745 ± 0.004 AB	25.42 ± 5.89 B	6.69 ± 2.08 B	0.48 ± 0.20 AB
NC358	11	0.766 ± 0.007 A	26.18 ± 3.67 B	16.14 ± 7.36 B	0.63 ± 0.25 AB

N/env denotes the number of RILs evaluated in each family per environment or the number of plots for each parental line per environment. Raw means and standard errors (SE) are reported, but ANOVA and pairwise *t*-tests were conducted on Box-Cox transformed data. Groups not connected by the same letter within each column are significantly different (pairwise two-tailed *t*-tests, *p* < 0.05). *** *p* < 0.0001 for ANOVA of trait in column header vs. family.

**Table 2:** Comparison of BDEN_inoc_, FER, FUM, and the ratio of FUM to FER (FUM:FER) under *F. verticillioides* inoculation among plots exhibiting distinct symptom types. Combined and family-specific analyses included (1) ANOVA for BDEN_inoc_, FER, FUM, and FUM:FER vs. symptom type, and (2) pairwise *t*-tests comparing BDEN_inoc_, FER, FUM, and FUM:FER between symptom types.

Family	Symptom type	BDEN_inoc_ (g mL^−1^)	FER (%)	FUM (ppm)	FUM:FER (ppm %^−1^)
Combined	Asym.	0.693 ± 0.003 A	19.58 ± 0.44 E	10.79 ± 1.69 A	0.96 ± 0.009 A
	Blush	0.682 ± 0.005 A	56.59 ± 1.88 C	15.51 ± 6.64 C	0.73 ± 0.017 C
	Starburst	0.656 ± 0.003 B	65.54 ± 0.75 B	18.40 ± 1.90 B	0.75 ± 0.008 C
	Purple	0.641 ± 0.003 C	50.51 ± 0.82 D	38.39 ± 4.75 BC	0.78 ± 0.009 B
	Moldy	0.576 ± 0.013 D	76.14 ± 2.64 A	97.66 ± 38.54 A	0.79 ± 0.038 BC
	ANOVA *p*	<0.0001 ***	<0.0001 ***	0.0002 **	<0.0001 ***
B73 ×	Asym.	0.713 ± 0.007 A	19.73 ± 1.36 C	4.58 ± 0.92 AB	0.91 ± 0.02 A
CML333	Blush	0.691 ± 0.005 B	58.68 ± 2.20 B	14.35 ± 5.01 AB	0.74 ± 0.02 B
	Starburst	0.685 ± 0.006 B	70.49 ± 1.49 A	13.80 ± 3.12 A	0.73 ± 0.02 B
	Purple	0.658 ± 0.006 C	54.04 ± 1.79 B	31.19 ± 10.31 B	0.71 ± 0.02 B
	Moldy	0.601 ± 0.021 D	77.95 ± 3.77 A	85.22 ± 53.51 AB	0.73 ± 0.07 B
	ANOVA *p*	<0.0001 ***	<0.0001 ***	0.0427 *	0.0001 **
B73 ×	Asym.	0.688 ± 0.005 A	18.09 ± 0.68 D	6.17 ± 0.70 A	0.94 ± 0.01 A
CML52	Blush	0.606 ± 0.020 B	46.78 ± 7.61 BC	8.75 ± 8.17 AB	0.70 ± 0.07 BC
	Starburst	0.660 ± 0.004 B	59.81 ± 1.32 B	10.46 ± 2.16 AB	0.74 ± 0.01 C
	Purple	0.655 ± 0.008 B	41.05 ± 1.57 C	21.21 ± 7.81 B	0.78 ± 0.02 B
	Moldy	0.503 ± 0.039 C	84.19 ± 5.38 A	32.09 ± 24.56 AB	0.66 ± 0.08 BC
	ANOVA *p*	<0.0001 ***	<0.0001 ***	0.113	<0.0001 ***
B73 ×	Asym.	0.693 ± 0.004 A	21.47 ± 0.79 C	14.70 ± 4.11 AB	0.99 ± 0.01 A
CML69	Blush	0.659 ± 0.016 AB	46.36 ± 6.61 B	39.96 ± 25.15 BC	0.78 ± 0.07 BC
	Starburst	0.657 ± 0.005 B	67.74 ± 1.40 A	21.59 ± 4.09 C	0.75 ± 0.02 C
	Purple	0.644 ± 0.006 B	54.22 ± 1.63 B	42.20 ± 8.61 C	0.78 ± 0.02 BC
	Moldy	0.588 ± 0.021 C	72.49 ± 5.74 A	179.86 ± 116.91 A	0.88 ± 0.07 AB
	ANOVA *p*	<0.0001 ***	<0.0001 ***	0.015 *	<0.0001 ***
B73 ×	Asym.	0.692 ± 0.006 A	20.88 ± 0.85 D	21.91 ± 7.24 A	0.97 ± 0.02 A
NC358	Blush	0.654 ± 0.016 AB	56.06 ± 4.60 BC	4.93 ± 1.84 B	0.67 ± 0.05 D
	Starburst	0.617 ± 0.006 B	66.45 ± 1.68 A	29.82 ± 5.56 A	0.77 ± 0.02 CD
	Purple	0.623 ± 0.006 B	51.19 ± 1.40 C	53.31 ± 10.22 A	0.82 ± 0.02 B
	Moldy	0.562 ± 0.029 C	68.74 ± 7.55 AB	60.22 ± 31.84 A	0.89 ± 0.08 ABC
	ANOVA *p*	<0.0001 ***	<0.0001 ***	0.0502	<0.0001 ***

ANOVA and pairwise *t*-tests were conducted on Box-Cox transformed data. Box-Cox-transformed means and standard errors (±) are reported. Groups not connected by the same letter within each column/family are significantly different (pairwise two-tailed *t*-tests, *p* < 0.05). ANOVA significance is denoted as * 0.05 > *p* ≥ 0.01; ** 0.01 > *p* ≥ 0.0001; *** *p* < 0.0001.

**Table 3:** Phenotypic and genotype-mean correlations among BDEN_inoc_, FER, FUM, and the ratio of FUM to FER (FUM:FER) under *F. verticillioides* inoculation within four NAM RIL families. Phenotypic correlations (r_P_) among plot-level phenotypes are in the upper diagonals. Correlations among genotype-means (r_G_) after controlling for genetic and field effects are in the lower diagonals.

B73 × CML333 Family				
r_P_/r_G_	BDEN_inoc_	FER	FUM	FUM:FER
**BDEN_inoc_**		−0.46 ***	−0.25 ***	−0.15 *
**FER**	−0.53 ***		0.17 **	−0.18 **
**FUM**	−0.31 **	0.28 ***		0.94 ***
**FUM:FER**	0.02	−0.33***	0.80 ***	
**B73 × CML52 family**				
**r_P_/r_G_**	**BDEN_inoc_**	**FER**	**FUM**	**FUM:FER**
**BDEN_inoc_**		−0.47 ***	−0.12 *	0.12 *
**FER**	−0.48 ***		0.08 ^ms^	−0.36 ***
**FUM**	−0.18 *	0.20 *		0.90 ***
**FUM:FER**	0.15 *	−0.46 ***	0.75 ***	
**B73 × CML69 family**				
**r_P_/r_G_**	**BDEN_inoc_**	**FER**	**FUM**	**FUM:FER**
**BDEN_inoc_**		−0.55 ***	−0.30 ***	0.02
**FER**	−0.53 ***		−0.01	−0.33 ***
**FUM**	−0.27 **	0.21**		0.95 ***
**FUM:FER**	0.06	−0.39 ***	0.81 ***	
**B73 × NC358 family**				
**r_P_/r_G_**	**BDEN_inoc_**	**FER**	**FUM**	**FUM:FER**
**BDEN_inoc_**		−0.59 ***	−0.19 **	0.11 ^ms^
**FER**	−0.58 ***		0.21 ***	−0.14 **
**FUM**	−0.25 **	0.12		0.94 ***
**FUM:FER**	0.15 ^ms^	−0.46 ***	0.79 ***	

Pearson correlation coefficients are reported in each cell, and significance is denoted as ^ms^ 0.1 > *p* ≥ 0.05 (marginally significant); * 0.05 > *p* ≥ 0.01; ** 0.01 > *p* ≥0.0001; *** *p* < 0.0001.

**Table 4:** Genotypic and field effect variance proportions, significance of days to silking (DTS), and broad-sense heritability of genotype-means (H) for BDEN_inoc_, FER, FUM, the ratio of FUM to FER (FUM:FER), and the absence/presence of five symptom types (asymptomatic, blush, starburst, purple, moldy) under *F. verticillioides* inoculation within four NAM RIL families. Mixed linear models were fit with genotype, environment (env), genotype*environment (G*E), block[environment] (B[E]) as random effects and DTS as a fixed effect.

Family	Trait	Random Effects (Variance Proportions)					Fixed Effect (*p*-Value)	H
		Genotype	Env.	G*E	B[E]	Error	DTS	
B73 × CML333	BDEN_inoc_	0.64	0.01	0.04	0	0.31	0.1	0.79
	FER	0.42	0.01	0.12	0	0.45	0.9	0.75
	FUM	0.02	0.61	0.16	0	0.21	0.3	0.12
	FUM:FER	0.03	0.58	0.10	0	0.29	0.6	0.17
	Asymptomatic	0.05	0.07	0.17	0.02	0.69	0.2	0.15
	Blush	0.18	0.13	0.30	0.01	0.38	0.004 **	0.44
	Starburst	0.12	0.18	0.17	0.01	0.53	0.9	0.33
	Purple	0.04	0.25	0.12	0.03	0.56	0.5	0.16
	Moldy	0.17	0.03	0.80	0	0.001	0.3	0.40
B73 × CML52	BDEN_inoc_	0.33	0.003	0	0.04	0.62	0.002 **	0.52
	FER	0.30	0.20	0.06	0.03	0.41	0.03 *	0.72
	FUM	0.03	0.46	0.01	0.04	0.46	0.1	0.17
	FUM:FER	0.02	0.48	0	0.01	0.48	0.3	0.12
	Asymptomatic	0.06	0.27	0.17	0.03	0.48	0.8	0.21
	Blush	0	0.01	0	0	0.99	0.04 *	0
	Starburst	0.11	0.28	0.18	0.02	0.42	0.6	0.35
	Purple	0.03	0.30	0.20	0.01	0.46	0.4	0.13
	Moldy	0.31	0.01	0.68	0	1 × 10^−5^	0.8	0.58
B73 × CML69	BDEN_inoc_	0.46	0.03	0.32	0.02	0.18	0.04 *	0.65
	FER	0.22	0.14	0.18	0.03	0.43	0.8	0.59
	FUM	0.02	0.70	0.13	0.01	0.14	0.1	0.15
	FUM:FER	0.02	0.70	0.07	0.02	0.19	0.3	0.21
	Asymptomatic	0.09	0.13	0.16	0	0.62	0.2	0.25
	Blush	0.06	0.04	0.01	0	0.88	0.05	0.18
	Starburst	0.08	0.24	0.15	0.003	0.53	0.005 **	0.27
	Purple	0.07	0.26	0.21	0.01	0.46	0.03*	0.24
	Moldy	0.17	0.03	0.80	0	8 × 10^−5^	0.009 **	0.39
B73 × NC358	BDEN_inoc_	0.23	0.08	0.56	0.02	0.12	0.07	0.40
	FER	0.27	0.22	0.06	0.04	0.40	0.003 **	0.70
	FUM	0.01	0.73	0.04	0	0.22	0.7	0.14
	FUM:FER	0.03	0.65	0.06	0	0.27	0.7	0.19
	Asymptomatic	0.05	0.04	0	0.05	0.86	0.08	0.15
	Blush	0.11	0.02	0.87	0	0.001	0.9	0.28
	Starburst	0.06	0.20	0.03	0.01	0.70	0.003 **	0.21
	Purple	0.07	0.25	0	0.01	0.67	0.03 *	0.24
	Moldy	0.13	0.01	0.85	0	0.002	0.4	0.32

Fixed effect *p*-value significance is denoted as: * 0.05 > *p* ≥ 0.01; ** 0.01 > *p* ≥ 0.0001.

**Table 5:** Physical positions (AGPv2) and marker effects of colocalized QTL. Marker effects were nested within family in the joint family stepwise regression model. As such, each colocalized QTL from the joint family analysis has four marker effects from the four non-B73 alleles (CML333, CML52, CML69, NC358) vs. the B73 allele. Colocalized QTL from the single-family analyses have one marker effect each (non-B73 parent allele vs. B73 allele).

Analysis	Chr.	Position (bp)	Associated Traits	Marker Effect on Trait (Non-B73 vs. B73 Allele)
Joint	1	17,256,105–	BDEN_inoc_	+0.003_CML333_; +0.013_CML52_; +0.019_CML69_; −0.001_NC358_
family		25,068,060	Blush	+0.054_CML333_; +0.002_CML52_; −0.001_CML69_; +0.007_NC358_
			CobLen	−4.874_CML333_; −3.899_CML52_; −5.097_CML69_; −13.611_NC358_
	2	20,081,914–	Blush	+0.073_CML333_; +0.001_CML52_; +0.012_CML69_; +0.005_NC358_
		50,634,516	CobDiam	+0.531_CML333_; +0.380_CML52_; +0.510_CML69_; +0.907_NC358_
			CobLen	+3.033_CML333_; +6.651_CML52_; +3.954_CML69_; +9.236_NC358_
			CobMass	+1.197_CML333_; +0.655_CML52_; +1.605_CML69_; +1.843_NC358_
			CobVol	+5.456_CML333_; +3.441_CML52_; +4.325_CML69;_ +8.825_NC358_
			Moldy	−0.026_CML333_; −0.013_CML52_; −0.021_CML69_; −0.006_NC358_
			Starburst	−0.001_CML333_; +0.023_CML52_; +0.044_CML69_; +0.047_NC358_
	2	193,400,572–	BDEN_inoc_	+0.025_CML333_; +0.003_CML52_; +0.010_CML69_; +0.002_NC358_
		196,813,641	CobMass	−1.545_CML333_; +0.418_CML52_; −1.314_CML69_; +0.220_NC358_
	5	205,258,663–	BDEN_inoc_	+0.009_CML333_; −0.016_CML52_; −0.002_CML69_; −0.016_NC358_
		208,297,027	Blush	−0.088_CML333_; −0.001_CML52_; −0.006_CML69_; −0.017_NC358_
			CobLen	−0.868_CML333_; −2.017_CML52_; −7.233_CML69_; −2.325_NC358_
			FER	−0.606_CML333_; +5.843_CML52_; −1.058_CML69_; +8.576_NC358_
	6	130,721,405–	Asym	+0.002_CML333_; +0.027_CML52_; +0.015_CML69_; +0.007_NC358_
		133,795,910	FER	−4.192_CML333_; −6.906_CML52_; −6.253_CML69_; −4.148_NC358_
	10	79,389,587–	CobDen	+0.005_CML333_; +0.012_CML52_; +0.0003_CML69_; +0.007_NC358_
		116,043,385	Starburst	−0.031_CML333_; −0.037_CML52_; −0.029_CML69_; −0.048_NC358_
B73 ×	1	19,128,807–	Blush	+0.052_CML333_
CML333		24,311,471	CobLen	−5.937_CML333_
	1	47,813,030–	FER	+6.660_CML333_
		57,347,172	FUM	+0.162_CML333_
	2	21,767,606–	BDEN_inoc_	+0.020_CML333_
		35,311,519	Blush	+0.065_CML333_
	7	4,823,956–	CobLen	+6.465_CML333_
		6,178,479	CobVol	+15.265_CML333_
			Starburst	+0.046_CML333_
	7	14,152,199–	FER	+4.985_CML333_
		23,748,237	FUM:FER	−0.009_CML333_
	8	130,983,626–	BDEN_inoc_	+0.015_CML333_
		151,773,181	CobDen	+0.010_CML333_
			CobDiam	−1.587_CML333_
			Starburst	+0.040_CML333_
B73 ×	5	83,007,601–	BDEN_inoc_	+0.018_CML52_
CML52		140,575,190	BDEN_uninoc_	+0.009_CML52_
	6	129,994,297–	Asym	+0.027_CML52_
		140,630,780	CobDiam	−0.744_CML52_
	6	154,530,689–	FER	−7.612_CML52_
		156,104,230	FUM:FER	+0.010_CML52_
	8	132,405,651–	BDEN_uninoc_	+0.010_CML52_
		146,365,593	CobVol	−6.924_CML52_
			FER	−5.718_CML52_
B73 ×	2	49,846,838–	CobMass	+2.016_CML69_
CML69		87,089,680	Moldy	−0.023_CML69_
			Starburst	+0.048_CML69_
	4	17,330,826–	BDEN_inoc_	−0.018_CML69_
		21,710,411	FER	+6.347_CML69_
	4	167,066,431–	Purple	+0.034_CML69_
		170,804,182	Starburst	−0.050_CML69_
	7	138,072,861–	Asym	+0.020_CML69_
		142,429,440	FER	−7.435_CML69_
B73 ×	2	144,159,847–	Purple	−0.031_NC358_
NC358		160,579,525	Starburst	+0.046_NC358_
	3	6,427,177–	BDEN_inoc_	+0.021_NC358_
		8,563,589	Starburst	−0.054_NC358_
	10	16,505,881–	Asym	+0.018_NC358_
		77,678,052	CobDen	+0.009_NC358_

## Supplementary Materials

The following are available online at http://www.mdpi.com/2072-6651/11/2/86/s1, Table S1: Genotype-means correlations among kernel bulk density (BDEN_inoc_), Fusarium ear rot (FER), fumonisin concentration (FUM), and the ratio of FUM to FER (FUM:FER) under *F. verticillioides* inoculation, and kernel bulk density (BDENuninoc) and cob density (CobDen), diameter (CobDiame), length (CobLen), mass (CobMass), and volume (CobVol) under uninoculated conditions within four maize RIL families.; Table S2: Summary of family-specific and joint family stepwise regression models and QTL for kernel bulk density (BDEN_inoc_), Fusarium ear rot (FER), fumonisin concentration (FUM), and the ratio of FUM to FER (FUM:FER) under *F. verticillioides* inoculation, and kernel bulk density (BDEN_uninoc_) and cob density (CobDen), diameter (CobDiam), length (CobLen), mass (CobMass), and volume (CobVol) under uninoculated conditions; Table S3: Physical positions (AGPv2) and associated traits of colocalized and trait-specific QTL from family-specific and joint family analyses.
