# Supplementary material for: Diverse Components of Resistance to Fusarium verticillioides Infection and Fumonisin Contamination in Four Maize Recombinant Inbred Families
Source: Toxins (Basel). 2019 Feb 1;11(2):86. doi: 10.3390/toxins11020086 (PMC6410224; doi:10.3390/toxins11020086)
Supplement: Supplementary file 1 [file toxins-11-00086-s001.zip › toxins-407715/Table_S1.docx]

Diverse Components of Resistance to *Fusarium verticillioides* Infection and Fumonisin Contamination in Four Maize Recombinant Inbred Line Families

**Laura Morales, Charles T. Zila, Danilo E. Moreta Mejía, Melissa Montoya Arbelaez, Peter J. Balint-Kurti, James B. Holland and Rebecca J. Nelson**

**Table 1.** Genotype-means correlations among kernel bulk density (BDEN_inoc_), Fusarium ear rot (FER), fumonisin concentration (FUM), and the ratio of FUM to FER (FUM:FER) under *F. verticillioides* inoculation, and kernel bulk density (BDEN_uninoc_) and cob density (CobDen), diameter (CobDiame), length (CobLen), mass (CobMass), and volume (CobVol) under uninoculated conditions within four maize RIL families.

| **Trait 1** | **Trait 2** | **B73 ×**  **CML333** | **B73 ×**  **CML52** | **B73 ×**  **CML69** | **B73 ×**  **NC358** |
| --- | --- | --- | --- | --- | --- |
| BDEN_uninoc_ | BDEN_inoc_ | 0.38*** | 0.42*** | 0.39*** | 0.37*** |
| BDEN_uninoc_ | FER | −0.09 | −0.32*** | −0.27** | −0.09 |
| BDEN_uninoc_ | FUM | 0.10 | 0.003 | −0.11 | −0.03 |
| BDEN_uninoc_ | FUM:FER | 0.14^ms^ | 0.23** | 0.05 | 0.02 |
| CobDen | BDEN_inoc_ | −0.02 | 0.08 | 0.12 | −0.03 |
| CobDen | BDEN_uninoc_ | 0.19** | 0.08 | 0.14^ms^ | 0.04 |
| CobDen | FER | 0.06 | −0.26** | −0.07 | −0.03 |
| CobDen | FUM | 0.05 | 0.01 | 0.05 | 0.09 |
| CobDen | FUM:FER | 0.01 | 0.16* | 0.10 | 0.13^ms^ |
| CobDiam | BDEN_inoc_ | −0.03 | −0.28** | −0.14^ms^ | −0.22** |
| CobDiam | BDEN_uninoc_ | −0.22** | −0.16* | −0.24** | −0.21** |
| CobDiam | CobDen | −0.13^ms^ | −0.16* | −0.40*** | −0.06 |
| CobDiam | FER | 0.01 | 0.28** | 0.16* | 0.20** |
| CobDiam | FUM | −0.01 | 0.08 | 0.03 | −0.11 |
| CobDiam | FUM:FER | −0.01 | −0.08 | −0.04 | −0.20** |
| CobLen | BDEN_inoc_ | 0.19* | 0.06 | −0.01 | −0.14^ms^ |
| CobLen | BDEN_uninoc_ | 0.01 | 0.12 | −0.11 | −0.07 |
| CobLen | CobDen | −0.15^ms^ | −0.18* | 0.04 | −0.17* |
| CobLen | CobDiam | 0.27** | 0.27** | 0.13^ms^ | 0.02 |
| CobLen | FER | −0.06 | 0.05 | 0.03 | 0.10 |
| CobLen | FUM | −0.07 | 0.08 | −0.004 | 0.08 |
| CobLen | FUM:FER | −0.05 | 0.03 | −0.006 | 0.004 |
| CobMass | BDEN_inoc_ | 0.06 | −0.13^ms^ | −0.07 | −0.30** |
| CobMass | BDEN_uninoc_ | −0.01 | −0.05 | −0.17* | −0.14^ms^ |
| CobMass | CobDen | 0.38*** | 0.33*** | 0.23** | 0.35*** |
| CobMass | CobDiam | 0.71*** | 0.69*** | 0.62*** | 0.72*** |
| CobMass | CobLen | 0.55*** | 0.53*** | 0.59*** | 0.38*** |
| CobMass | FER | 0.04 | 0.10 | 0.09 | 0.23** |
| CobMass | FUM | −0.002 | 0.13^ms^ | 0.03 | −0.01 |
| CobMass | FUM:FER | −0.04 | 0.07 | 0.004 | −0.13^ms^ |
| CobVol | BDEN_inoc_ | 0.09 | −0.18* | −0.15^ms^ | −0.31** |
| CobVol | BDEN_uninoc_ | −0.11 | −0.09 | −0.23** | −0.17* |
| CobVol | CobDen | −0.20** | −0.30*** | −0.36*** | −0.19* |
| CobVol | CobDiam | 0.82*** | 0.79*** | 0.82*** | 0.78*** |
| CobVol | CobLen | 0.68*** | 0.66*** | 0.54*** | 0.51*** |
| CobVol | CobMass | 0.82*** | 0.79*** | 0.82*** | 0.84*** |
| CobVol | FER | −0.003 | 0.25** | 0.11 | 0.25** |
| CobVol | FUM | −0.03 | 0.11 | 0.01 | −0.06 |
| CobVol | FUM:FER | −0.04 | −0.04 | −0.04 | −0.21** |
| FER | BDEN_inoc_ | −0.53*** | −0.48*** | −0.53*** | −0.58*** |
| FUM | BDEN_inoc_ | −0.31** | −0.18* | −0.27** | −0.25** |
| FUM | FER | 0.28*** | 0.19** | 0.21** | 0.12 |
| FUM:FER | BDEN_inoc_ | 0.02 | 0.15* | 0.06 | 0.15^ms^ |
| FUM:FER | FER | −0.33*** | −0.46*** | −0.39*** | −0.46*** |
| FUM:FER | FUM | 0.80*** | 0.75*** | 0.81*** | 0.79*** |

Pearson correlation coefficients are reported in each cell, and significance is denoted as ^ms^0.1 > *p* ≥ 0.05 (marginally significant); *0.05 > *p* ≥ 0.01; **0.01 > *p* ≥ 0.0001; ****p* < 0.0001.
